# Efficacy and design requirements of UV light cabinets for disinfection of exchangeable non-sterilizable “dental objects”

**DOI:** 10.1038/s41598-023-45481-w

**Published:** 2023-11-13

**Authors:** M. A. Moufti, M. Hamad, A. Al Shawa, A. Mardini, S. Ghebeh

**Affiliations:** 1 College of Dental Medicine, University of Sharjah, P.O.Box 27272, Sharjah, UAE; 2 College of Health Sciences, University of Sharjah, Sharjah, UAE; 3 Research Institute for Medical and Health Sciences, University of Sharjah, Sharjah, UAE

**Keywords:** Microbiology techniques, Infection control in dentistry

## Abstract

Non-sterilizable items such as prosthodontics items constitute a high risk of transmitting dangerous pathogens, including Coronavirus, between patients and healthcare personnel. Although UV rays are recognized for their germicidal efficacy, large and expensive UV devices previously hindered their adoption in dental offices. During the COVID-19 pandemic, small UV devices became available for domestic use, albeit with varying designs and effectiveness. Our study assesses the disinfection capacity of a UV light cabinet for four dental materials and discusses crucial design features for effective performance. Specimens of each material (silicone impressions, stone cast, acrylic denture base, and indelible pencils) were contaminated with *Escherichia coli* Bl21, and randomly divided into three study groups: UV device (UVG), impressions disinfection solution (SG), and control (CG). The experiment was repeated thrice, and disinfection efficacy assessed by colony forming units (CFU) count. A 2.5-min UV exposure achieved full disinfection for all materials. Significantly different results were found between groups (p < 0.05, one-way ANOVA, Tukey HSD), except for indelible pencils, where UVG and SG were both highly effective. UV cabinets surpass SG’s disinfection efficacy. Compact UV devices can offer affordable, portable, and efficient disinfection for non-sterilizable dental objects, with careful consideration of wavelength, exposure, intensity, and safety.

## Introduction

Dentistry is one of the healthcare professions with the highest potential for contamination with patients’ fluids and for transmitting healthcare-associated infections (HAIs)^1^. It is for this reason why, at the beginning of the COVID-19 pandemic, dental practices in many parts of the world closed down voluntarily or by government-imposed law, except for the management of emergency cases and under exceptional precautions^2^. Disinfection protocols in dental clinics have been tightened but remained widely concerning personal protecting equipment (PPE), surface sanitization, and distancing^3–5^.

Dental patients and healthcare workers are typically at risk of acquiring infectious pathogens due to exchanging objects and materials between the dental clinic and laboratory, including the SARS-CoV-2, HIV, Hepatitis A, B, and C viruses^6^, *Pseudomonas aeruginosa*, and *Staphylococcus aureus*.

While sterilization by heat, wet or dry, is the gold standard, chemical sterilization is sometimes needed in dental settings for the decontamination of heat- or moisture-sensitive instruments or equipment that cannot withstand autoclaving^7^. Chemical disinfection, however, is technique sensitive and is known to have a detrimental effect on rubber- or plastic-based dental instruments^8^.

Complete immersion of hydrocolloid and polyether impressions in a disinfectant solution provides better exposure but may also induce dimensional changes owing to imbibition, hence, inducing dimensional inaccuracies^9^. Similarly, immersion of gypsum or die stones in disinfectant solutions may induce dimensional changes and affect setting time^10^. For this reason, dental impressions are routinely disinfected by spraying without affecting their dimensional accuracy. However, the technique is prone to human error as it does not ensure reaching all the hidden surfaces and undercuts, thereby increasing the risk of cross-contamination^10^. Furthermore, improper use of chemicals, inadequate contact times, and application techniques can all contribute to incomplete disinfection and the spreading of pathogens^11^. Ultraviolet (UV) radiation has the ability to reach all surfaces by reflecting light in all directions, and hence, it might be a better alternative for disinfection in dental settings.

Furthermore, UV rays have long been known as efficient bactericidal properties due to their potential to cause DNA damage and cessation of bacterial reproduction^12,13^. Therefore, UV radiation can be used as an alternative to heat- and chemical-based disinfection procedures^14^. Besides, the International Ultraviolet Association has reported that UV disinfection may also prove beneficial in reducing the transmission of COVD-19^15^. However, UV light works only on surfaces within the field of view, and its strength decreases with distance^16^. Moreover, low dosages do not effectively inactivate some viruses and spores^13^.

UV hoods devices are considered the gold standard method for disinfection in microbiology laboratories^17^. However, given their large size and cost, these devices were not popular in dental practices, and disinfection had been routinely carried out using disinfecting solutions, both by spraying and immersion^18^. Recently, the surge in public awareness of the importance of infectioncontrol has led to the thriving of manufacturing UV devices for personal use^19,20^. These devices have become available in various smaller sizes and at relatively low costs, which made them a viable and effective alternative to chemicals in dental practice^21,22^. However, many of these did not possess the appropriate UV wavelength, intensity, or design to shine the UV radiation on all aspects of the object to be disinfected^20^. The use of smaller-sized UV devices with the right UV wavelength and intensity can offer reliable, cost-effective, and time-efficient disinfection options in dental settings^22^.

This research paper aims to test the UV light’s disinfection efficacy of four dental-related objects, silicone putty impressions, stone casts, acrylic denture bases, and indelible pencils compared to the current practice of spraying a disincentive solution.

## Materials and methods

**Figure 1:**
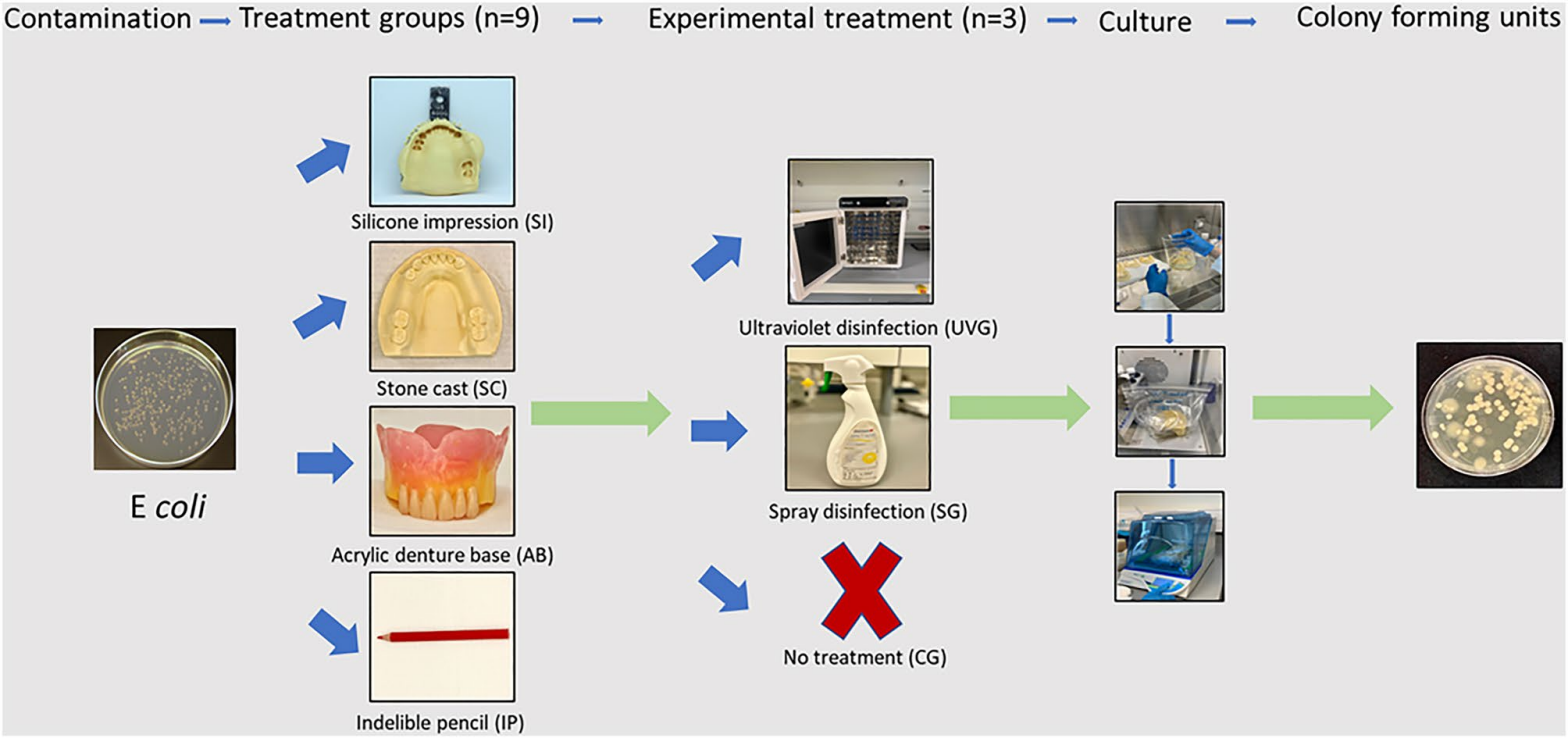
Flowchart of the steps involved in testing and the experimental materials used.

The experimental in vitro study was carried out at the College of Dental Medicine and the Research Institute of Medical & Health Sciences at the University of Sharjah. The study groups comprised the UV light group (UVG), the spray group (SG), and a negative control group (CG) where no disinfection was performed (Fig. 1). The disinfection was tested on four materials of different characteristics: Silicone putty impressions (SI), stone cast (SC), acrylic denture base (AB), and indelible pencils (IP). Nine specimens from each material were made for each study group, and the experiment was repeated on three different days.

### Microbial inoculum preparation and contamination

The specimens were contaminated with *Escherichia coli* (*E. coli*) strain BL21^23^. The contamination solution was prepared to a concentration of 0.5 McFarland. Using an aseptic technique, 20 ml of an aliquot of 0.5 McFarland *E. coli* solution was transferred into a sterile glass beaker containing 200 ml of sterile phosphate buffer saline (PBS) (Sigma Aldrich, Germany). The specimens were fully immersed in the diluted *E. coli* stock solution and were then incubated for 2 min at 37 °C to dry. After completion of the drying period, the contaminated samples were divided randomly into three groups, each of which was subject to one of the disinfection methods.

### Disinfection

The first group of specimens was placed in a UV light chamber for an exposure time of 2.5 min (150 s). The germicidal disinfection cabinet (MADA, Model No.: MA20-16W) was designed and manufactured in United Arab Emirates, UAE, and assembled in China. The device is equipped with four germicidal lamps (Philips, Poland) with a wavelength of 253.7 nm and an intensity of 1024 μW/cm^2^. The lamps were distributed: two on the top side and two on the bottom side, and the interior design of the chamber was composed of mirror walls to reflect the UV rays. A UV flux sensor (Model SDL470, EXTECH USA) exclusively designed for UVC light detection was employed to assess the intensity of UVC irradiation within the cabinet. This recorded a maximum UVC intensity of 1024 μW/cm^2^ which was attained after 2 min of irradiation.

The second group (positive control) was disinfected using Zeta 7 spray (Zhermack, Italy). The solution is composed of 83 g ethanol, 10 g 2-propanol, non-ionic surfactants, additives, auxiliaries, and water to 100 g, with a distance of 10 cm to ensure maximum coverage/exposure. They were then allowed to evaporate for 3 min. The third group received no treatment and acted as a negative control. The experiment was repeated three times to ensure the reproducibility of the results.

### Culture

After exposure, the specimens were placed in small to medium-sized zip lock bags and washed with 50 ml of PBS. The suspension was placed in a shaking incubator for 1 min. This allowed the suspension of the microorganism in the solution. Next, an amount of 20 ml of the PBS of each sample was aseptically plated into Mueller Hinton agar media by pouring plate method for the observation and counting of colonies. The media plates were incubated at 37 °C for 24 h.

Colony Forming Units (CFUs) were counted for each of the four materials, and compared among the three groups (one-way ANOVA, and a post-hoc Tukey test, p = 0.05) using the GraphPad software.

## Results

**Figure 2:**
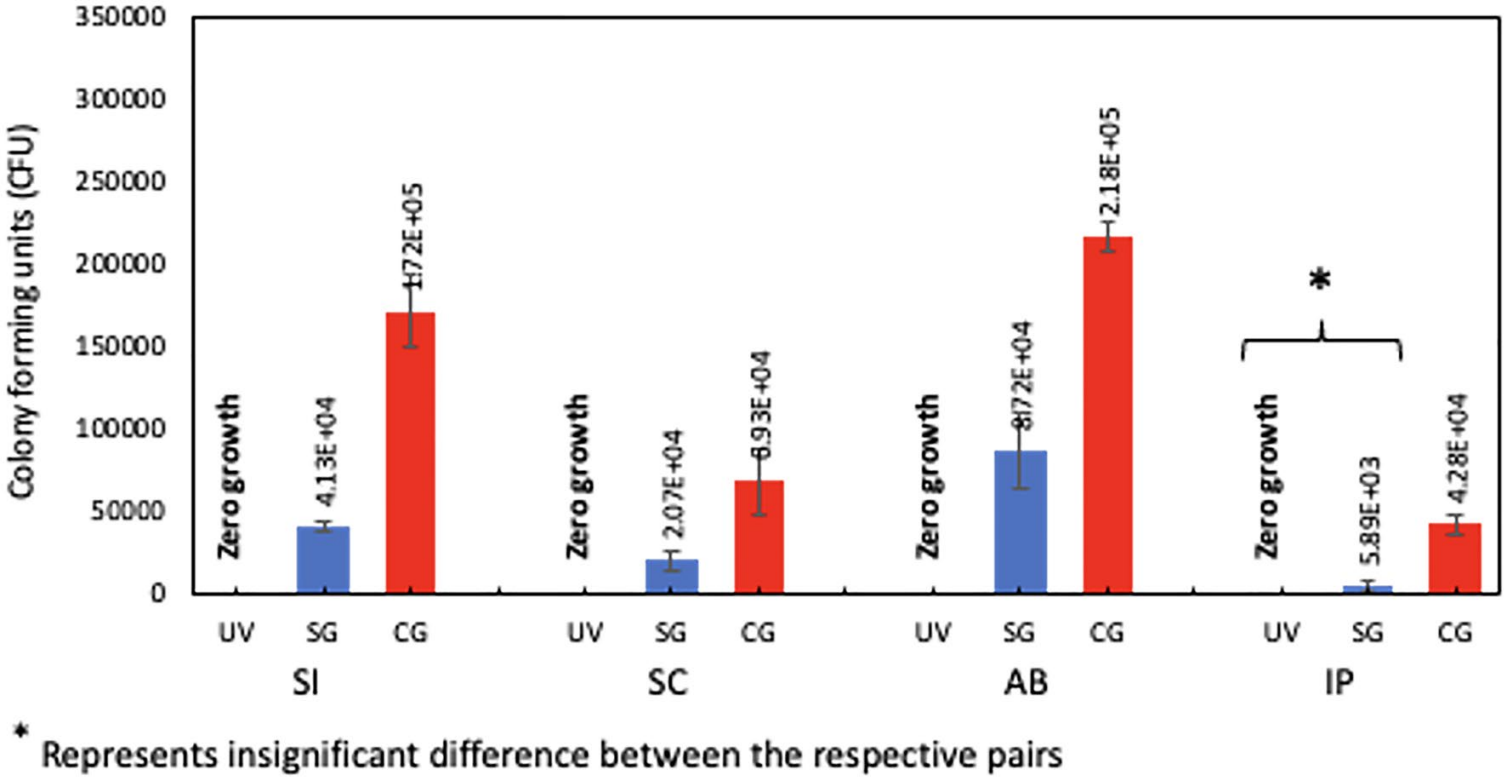
Mean (± SD) CFU values for SI, SC, AB, IP groups that were disinfected using different techniques.

**Figure 3:**
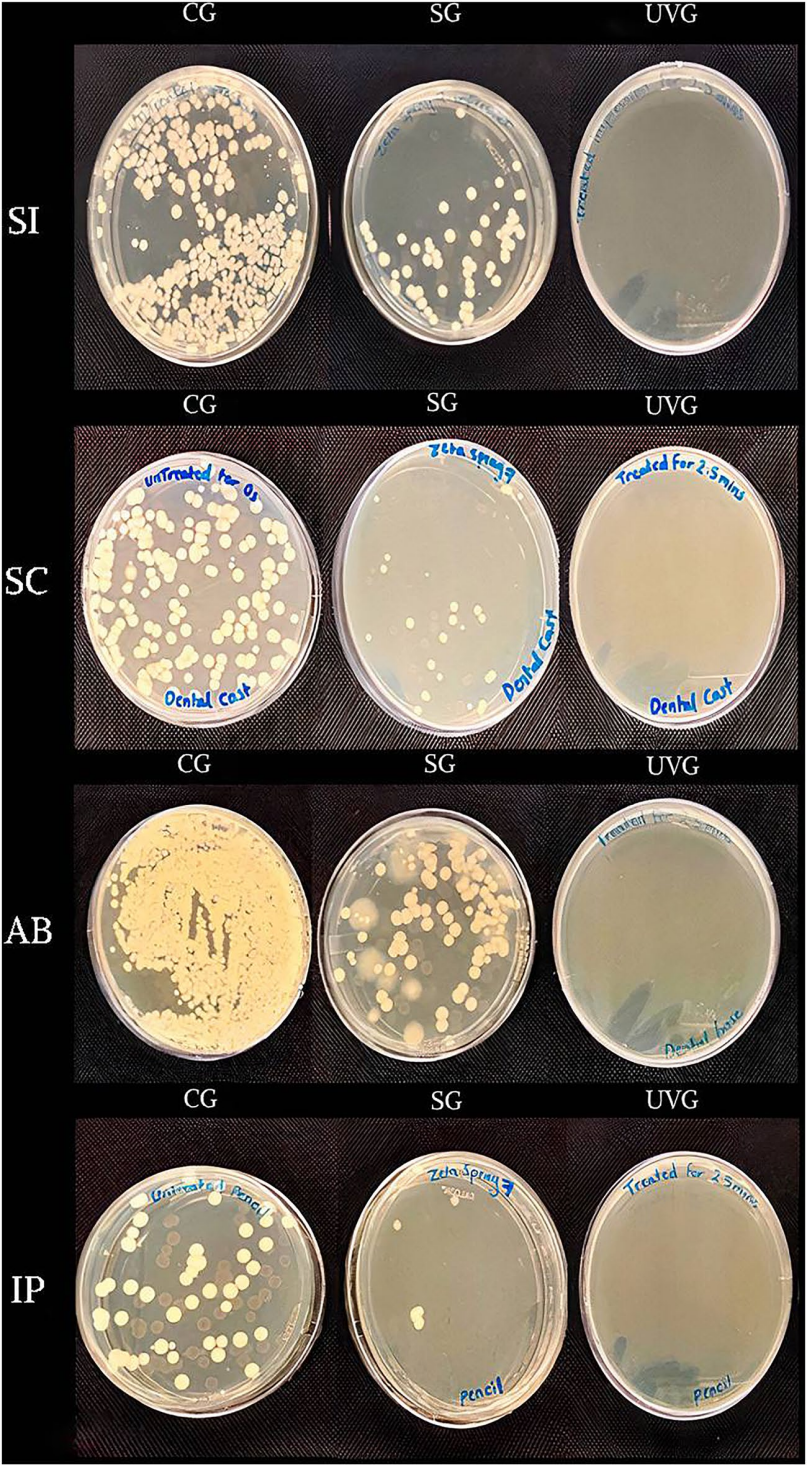
Agar plates showing bacterial growth following disinfection of SI, SC, AB, and IP previously contaminated with *E. coli*, using different disinfection techniques.

As can be seen in Figs. 2 and 3, the UV group (UVG) has achieved a complete eradication of the tested organism (CFU = 0) for all materials, while the spray group (SG) has eliminated most microbial colonies. The one-way ANOVA test showed a significant difference between the mean CFU counts for all the materials against all the treatment groups (p < 0.05) (Table 1). Similarly, the post-hoc Tukey test showed a significant mean CFU difference between all possible treatment groups, except the UVG vs. SG (Table 2).

**Table 1:** Comparison of the mean CFUs between the three disinfection methods (ANOVA).

Object	Disinfection method	Mean CFU	Standard deviation	F (df)^±^	p value
Silicone impression (SI)	Untreated	171,833.33	21,673.72	152.578 (2)	< 0.001*
	UV	0.00	0.00		
	Zeta 7 spray	41,333.33	2179.45		
Stone cast (SC)	Untreated	69,333.33	20,025.68	25.950 (2)	0.001*
	UV	0.00	0.00		
	Zeta 7 spray	20,722.22	6185.41		
Acrylic base (AB)	Untreated	217,611.11	8866.81	178.686 (2)	< 0.001*
	UV	0.00	0.00		
	Zeta 7 spray	87,166.67	22,924.39		
Indelible pen (IP)	Untreated	42,777.78	5058.91	147.645 (2)	< 0.001*
	UV	0.00	0.00		
	Zeta 7 spray	5888.89	2678.79		

*p < 0.05, significant.

^±^F Statistic ANOVA (degree of freedom).

**Table 2:** Pairwise comparison of the mean CFUs between the three groups (Post-hoc Tukey—ANOVA).

Object	Pair-wise comparison	Mean difference of CFU	p value	95% confidence interval	
				Lower bound	Upper bound
PVS impression	Untreated—Zeta 7	130,500*	< 0.001*	105,907	155,093
	Untreated—UV	171,833*	< 0.001*	147,240	196,426
	Zeta 7—UV	41,333*	< 0.001*	16,740	65,926
Stone cast (SC)	Untreated—Zeta 7	48,611*	0.006*	34,913	62,310
	Untreated—UV	69,333*	< 0.001*	55,635	83,032
	Zeta 7—UV	20,722*	0.00256*	7024	34,421
Acrylic base (AB)	Untreated—Zeta 7	130,444*	< 0.001*	87,358	173,530
	Untreated—UV	217,611*	< 0.001*	174,525	260,697
	Zeta 7—UV	87,167*	0.001*	44,081	130,253
Indelible pen (IP)	Untreated—Zeta 7	36,889*	< 0.001*	29,227	44,551
	Untreated—UV	42,778*	< 0.001*	35,116	50,440
	Zeta 7—UV	5889	0.153	− 1773	13,551

*p < 0.05, significant.

## Discussion

In the wake of the COVID-19 pandemic that resulted with forceful closure of dental practices due to cross-contamination concerns, we aimed to reassess the disinfection efficacy of common non-sterilizable dental materials. These items, widely exchanged in dental settings, pose a significant cross-contamination risk among patients, dentists, and lab technicians. Our study included indelible pencils, chosen for their permanence in marking dentures and labeling impressions. Despite their single-use nature, some clinics reuse them, warranting attention to this issue.

Our results demonstrate that the UV irradiation group (UVG) achieved a marked and consistent complete eradication of *E. coli*, as evidenced by a colony-forming unit (CFU) count of 0 across all materials (Fig. 2). On the other hand, the spray treatment group (SG) displayed effective microbial reduction, although with residual colonies present.

UV light disinfection is highly effective against diverse microorganisms, safe, and cost-effective, reducing aerosol spread compared to chemicals. Handheld UV wands work well but may harm eyes without proper distance control^24^. UV chambers with eye-protective doors and safety features are now accessible to dental facilities. However, commercially available devices differ in design and equipment, so selecting one with suitable features is crucial for effective disinfection.

Effectiveness of the UV disinfection unit is dependent on multiple parameters, including the UV radiation wavelength and intensity, the design of the UV chamber, the distance between the UV machine's bulb and the specimen, and the time of exposure to the radiation. Based on wavelength, there are three types of UV rays: UV-A (320–400 nm), UV-B (280–320 nm), and UV-C (100–280 nm). UV-C radiation has the most bactericidal effect and was chosen in our study^25^. The main bactericidal effect of UV irradiation is the result of photoproducts. When DNA absorbs UVC radiation, pyrimidine dimer production causes nucleic acid damage, which leads to bacterial cell death^25^.

The importance of light intensity is well established. Previous research showed that UV exposure of 24 Watt (3750 μW/cm^2^) for 90 s eradicates Candida albicans^26^. Dental impressions contaminated with HBV and HIV were UV-disinfected in 30 s only, but with the use of very high intensity (7000 μW/cm^2^)^27^. In our study, 100% bacteria eradication was achieved using four germicidal lamps, two on the upper and two on the lower walls, each with a 16W lamp producing 1024 μW/cm^2^. This was achieved in less than 3 min, the time recommended for disinfection with spray^26,27^.

Direct access of the disinfectant to microorganisms is essential to ensure disinfection of all surfaces. Lualdi et al.^28^ and Andersen et al.^29^ studied the effect of UVC lamps in hospital settings and concluded that the appropriate position of UV lamps or the presence of a UV reflector allows direct penetration to the microorganism is required to achieve complete disinfection. Furthermore, the intensity of light, UV-C included, decreases with the square of the distance due to the spreading of light over a larger area^11^. In this study, reflective walls ensured adequate exposure for all surfaces and micro-porosities. This explains the low CFU count in the indelible pencils (IP) group, as their smooth surface facilitated uniform disinfection. UV disinfection offers a safe way to reuse indelible pencils in dental settings, despite initial discouragement due to cross-contamination concerns.

The UV exposure times vary significantly in the literature, ranging from 30 s to 20 min for various materials and under different lamp intensities and exposure conditions^6,26,27,30,31^. In our study, we tested UV exposure durations of 30 s, 2.5 min, and 5 min. Complete bacteria elimination was achieved at just 2.5 min, towards the lower end of the above time range. This is likely because of the strategically placed UV bulbs and use of reflective walls ensuring uniform exposure.

The required sample size was estimated based on the desired effect size. The aim of this study was to establish if the UV method has a significantly better antibacterial effect than current practice (spraying) to the extent that would warrant change in practice. An 80% reduction in the CFU would be considered a sufficiently large effect size. The required sample size was calculated using G*Power to achieve a power of 95% for one-way ANOVA between three groups of equal allocation (i.e., UV, Spray, and Control) and an effect size of 80%. The calculation identified that a sample size of 9 (3 in each group) would achieve a power of 99.6%.

Since our study would be able to statistically detect the required “clinically significant” effect size with a sample of nine at a sufficient at a “high” power, increasing the sample size to detect a smaller and “statistically significant but non-clinically significant” difference would be counterproductive. However, in order to ensure reproducibility and independence of observations, we obtained three technical replicates for each experiment, and repeated the experiment on three different days to achieve biological replicates.

The negative control group confirmed the necessity of using disinfectants. This aligns with Rossi’s findings^32^, indicating UV-C’s superiority over 70% alcohol for disinfecting elastic bands^33^. Other studies reported microbial growth after using ethanol- and di Didecyldimethylammonium chloride-based sprays^34^. Resendiz et al.^35^ also demonstrated UV-C outperforming isopropanol-based spray against anaerobes. In contrast, André et al.^36^ found no significant difference between UV light and 70% ethanol for 1-min exposure. The difference between these results and ours may stem from methodological variations, such as full immersion in ethanol by André while the current study employed the spray disinfection technique commonly used in clinical dentistry. Umezawa et al.^37^ demonstrated similar disinfection effects between a portable UV device and ethanol wipes.

In conclusion, our study highlights the differing effectiveness of UV irradiation and spray treatment in eliminating microbes from various materials, with statistically significant differences confirmed through effect size and power analyses. While UV irradiation effectively eradicated microbes in our study, it is advisable to pre-clean dental materials to ensure optimal UVC light penetration. Future research should explore additional materials, pathogenic microbes, and compare to standardized sodium hypochlorite immersion for further validation^38,39^. These findings lay the groundwork for enhanced disinfection strategies in dental practice, bolstering infection control and public health.

### Study limitations

The study has opted to examine which of the tested methods (chemical solutions and UV) provides better “accessibility” and exposure to items with complex surface design such as dental impressions and models. *E. coli* was used as the test organism since it has been studied extensively in the past, which facilitates comparability with other studies in the field. Additionally, submergence disinfection, which is a well-established method of impression sterilization, was not used and should be explored in future studies. Future studies are needed to test the efficacy on other dental-related microbes, including the Coronavirus, and to measure the cost-effectiveness and effects on the dimensional stability of UV machines.

## Conclusion

The results of this study provide valuable insights into the efficacy of UV light and an impression disinfection solution in the disinfection of four dental materials. A complete eradiation of the organisms was attained in the UV group after exposure to UV light for 2.5 min, underscoring its reliability and potency. With the adherence to proper design, operational protocols, and safety measures, cost-effective UV sterilization devices stand out as promising disinfection methods in the field of dentistry.

## Data Availability

All data generated or analyzed during this study are included in this published article.
